# Cellular mechanisms of incretin hormone secretion

**DOI:** 10.1530/JME-23-0112

**Published:** 2024-02-22

**Authors:** Marta Santos-Hernández, Frank Reimann, Fiona M Gribble

**Affiliations:** 1 Institute of Metabolic Science, Addenbrooke’s Hospital, Cambridge, UK

**Keywords:** incretin, GLP-1, GIP, enteroendocrine cells, diabetes, obesity

## Abstract

Enteroendocrine cells located along the gastrointestinal epithelium sense different nutrients/luminal contents that trigger the secretion of a variety of gut hormones with different roles in glucose homeostasis and appetite regulation. The incretin hormones glucagon-like peptide-1 (GLP-1) and glucose-dependent insulinotropic polypeptide (GIP) are involved in the regulation of insulin secretion, appetite, food intake and body weight after their nutrient-induced secretion from the gut. GLP-1 mimetics have been developed and used in the treatment of type 2 diabetes mellitus and obesity. Modulating the release of endogenous intestinal hormones may be a promising approach for the treatment of obesity and type 2 diabetes without surgery. For that reason, current understanding of the cellular mechanisms underlying intestinal hormone secretion will be the focus of this review. The mechanisms controlling hormone secretion depend on the nature of the stimulus, involving a variety of signalling pathways including ion channels, nutrient transporters and G-protein-coupled receptors.

## Introduction

The enteroendocrine system regulates diverse physiological and homeostatic gastrointestinal (GI) functions that enable the body to respond appropriately to feeding and fasting. Enteroendocrine cells (EECs) represent around 1% of the gut epithelial cell population and are distributed along the GI tract from the stomach to the rectum, together forming the largest endocrine organ in the body (Ahlman & Nilsson 2001). They secrete over 20 different hormones involved in metabolic processes related to digestion, intestinal motility, gastric emptying, glucose homeostasis and appetite regulation. The circulating gut hormone profile at any given time reflects what we have eaten recently and when we last ate, providing up to date information about dietary nutrients entering the bloodstream. The incretin hormones glucose-dependent insulinotropic polypeptide (GIP) and glucagon-like peptide-1 (GLP-1) play a particularly important role in glucose metabolism and regulation of food intake and body weight, as evident from their development as therapies for type 2 diabetes and obesity (Campbell *et al.* 2023).

EECs have been traditionally categorised into distinct cell types based on their morphology and the main secretory hormones they produce. Thus, GIP is secreted by EECs known as K-cells, GLP-1 and peptide YY (PYY) by L-cells, and cholecystokinin (CCK) by I-cells. However, many studies have demonstrated co-localisation and co-expression of hormones within the same EEC cell-type by immunohistochemistry (Mortensen *et al.* 2003) and transcriptomic analysis (Haber *et al.* 2017). Mouse intestinal cells expressing *Gcg* (encoding GLP-1) or *Gip*, for example, also exhibited mRNA expression and immunostaining for CCK, GIP and secretin (SCT) (Habib *et al.* 2012), and *Cck*-labelled cells from mouse duodenum had high mRNA levels for *Cck*, *Sct*, *Gip* and *ghrelin*, as well as some *Gcg* and *Pyy* (Egerod *et al.* 2012). Cluster analysis of single EEC RNA sequencing data have similarly found high levels of overlap between cells expressing *Gcg*, *Gip* and *Cck* (Haber *et al.* 2017, Bai *et al.* 2022, Hayashi *et al.* 2023), suggesting that the cellular mechanisms underlying release of these hormones should be considered in parallel. Despite the overlapping hormonal expression profiles observed in individual EECs, *in vivo* hormonal responses differ with respect to preferred nutrient stimuli. This might reflect the distribution of hormones along the length of the intestine relative to variations in pH and sites of nutrient absorption, or predominant expression of hormones in separate cells expressing different nutrient sensors.

Each hormone has a distinct distribution along the gut and between species (Roberts *et al.* 2019). Some hormones, like serotonin, are produced along the whole GI tract, whereas others are restricted to a particular location, with GIP and CCK being found mainly in the proximal small intestine, and GLP-1 and PYY more in the distal small intestine and colon in humans. A region-specific profile of incretin responses to nutrients has been demonstrated in human participants by intraluminal perfusion studies directing glucose to either the proximal or distal small intestine (Zhang *et al.* 2019, 2022); GIP-responses were bigger in response to duodenal compared with ileal glucose infusion and the opposite was true for GLP-1, and it was concluded that proximally delivered glucose only stimulated substantial GLP-1 responses when the absorptive capacity of the proximal intestine was exceeded. Interestingly, intraduodenal administration of nutrient-free hyperosmolar saline solutions (1500 mOsm/L) significantly increased human plasma CCK, GLP-1, PYY and neurotensin but not GIP or motilin levels (Veedfald *et al.* 2018), pointing towards a release of more distally expressed hormones by this stimulus, with the underlying sensory mechanism currently unclear.

Within an individual EEC, there has been some debate about whether each hormone is packaged into its own distinct vesicular pool or if individual vesicles contain mixtures of hormones. Some studies using immunohistochemistry and confocal microscopy suggested that peptide hormones may be located in separate storage vesicles (Grunddal *et al.* 2016, Fothergill *et al.* 2017), but more detailed analysis by super-resolution microscopy of EECs producing INSL5, GLP-1 and PYY revealed that in mouse and human the three hormones are located together in >80% of individual vesicles, and in murine primary cultures they are secreted in parallel in response to a range of stimuli (Billing *et al.* 2018). Quantification of immunohistochemically stained tissues can be inaccurate due to a lack of specificity and sensitivity of the antibodies, and the methods used for image analysis. However, as EECs are known to modify their hormone expression during maturation (Beumer *et al.* 2018), individual vesicles may contain different hormones because they were generated at different times, reflecting the temporal expression history of the single cell. Preferential responsiveness to selective stimuli might thus reflect the maturity and position of EECs along the crypt-villus axis, but rather less is known about receptor location along this axis, partially due to a lack of suitable antibodies.

## Models to study sensing mechanisms

Studies to investigate mechanisms underlying the function of EECs have employed a variety of systems, including *in vitro* models (cell lines, primary epithelial cultures, intestinal organoids), *ex vivo* models (Ussing chambers, isolated perfused intestine), *in vivo* studies in animals, and human studies. Benefits and limitations of these models are outlined below and have also been discussed elsewhere (Goldspink *et al.* 2018*a*, Kuhre *et al.* 2021)

The three most widely used cell lines to study incretin hormone secretion and enteroendocrine cell signalling pathways are STC-1, GLUTag and NCI-H716. STC-1 cells were developed as a murine model of EECs from the upper GI tract and are used to study the secretion of CCK, secretin, GIP and GLP-1 (Rindi *et al.* 1990). The plurihormonal nature of STC-1 cells led to some concern about the validity of this cell line, but this seems to represent the overlapping nature of native EECs from the upper GI tract, and in functional studies the cell line generates secretory responses to a range of physiological stimuli. GLUTag is a cell line derived from an SV40-large T-antigen-expressing tumour of the mouse large intestine (Brubaker *et al.* 1998), and produces CCK, GLP-1 and neurotensin (Kuhre *et al.* 2016). It faithfully reproduces many of the features of GLP-1 and CCK secretion observed in physiological studies, and has been widely used to study enteroendocrine signalling pathways. NCI-H716 is a human GLP-1 secreting cell line derived from a poorly differentiated caecal adenocarcinoma (Reimer *et al.* 2001), which is grown in suspension culture prior to seeding in wells for experimentation. It offers the benefit of being a human cell line but is less well characterised and validated than the murine cell lines. Although enteroendocrine cell lines exhibit many similarities to native EECs, they are simplified models without adjacent cell types (enterocyte, Paneth, goblet), and have altered physiological cell morphology and hormone processing characteristics compared with native L-cells (Kuhre *et al.* 2016).

Primary intestinal epithelial cultures from adult mouse and human tissues have been developed to study incretin hormone secretion and signalling (Reimann *et al.* 2008, Habib *et al.* 2013). When used in combination with transgenic mouse models expressing fluorescent labels in defined EEC populations, they have allowed for the identification of living EECs for single-cell functional analysis, enabling an interdisciplinary approach to EEC function combining techniques such as transcriptomics, live-cell second messenger imaging, electrophysiology and pharmacology. Primary cultures are particularly suitable for measuring acute or short-term responses but are not useful for studying cell development and differentiation as they survive for up to 2 weeks in standard tissue culture media but do not generate new EECs (Reimann *et al.* 2008). Some laboratories have also used acutely isolated intestinal biopsies or mucosal isolates for secretion experiments (Sun *et al.* 2017), but in our hands the results of such preparations were more variable than with cultured cells.

Intestinal organoids are increasingly used as three-dimensional renewable epithelial cultures which maintain a polarised epithelium and generate different types of EEC. They are usually created from fresh intestinal tissue biopsies containing crypt stem cells and exhibit regional identity depending on the site of origin of the original donor sample (Beumer *et al.* 2020). Techniques to generate intestinal organoids containing functional EECs from induced pluripotent stem cells (iPSC) are improving (Sanchez *et al.* 2022), but further validation is needed to confirm that the EECs in iPSC-derived organoids mirror the functional and transcriptomic characteristics of native EECs. One particular advantage of organoid cultures is their capacity to be genetically modified – a feature that has seen the production in recent years of human organoid models carrying fluorescent markers in different EEC types for transcriptomic and live cell functional studies (Beumer *et al.* 2020, Goldspink *et al.* 2020). Organoid maintenance requires additional factors in the media to replicate the stem cell niche and to generate EECs that faithfully reproduce many of the physiological features of native tissues and model cell lines. It has been suggested that organoids may retain some metabolic characteristics of the original donor (Beyaz *et al.* 2016), but with prolonged time in culture it is likely that the highly controlled conditions needed for organoid growth and differentiation would reverse some or all of the impacts of donor metabolic conditions such as obesity or diabetes.

Culturing organoids in transwells exposed to an air-liquid interphase has enabled generation of polarised intestinal epithelia containing GLP-1 producing cells (Villegas-Novoa *et al.* 2022). These allow directional application of stimuli, critical for translational approaches to target EECs with small molecules, because of the potential opportunities to develop non-absorbable ligands with minimal systemic side effects, provided that their target receptors are readily accessible from the gut lumen. *Ex vivo* preparations such as Ussing chambers (Brighton *et al.* 2015) and vascular perfused intestinal models (Modvig *et al.* 2021) also maintain the polarity of the epithelial layer and enable application of stimuli to the apical or basolateral compartments.

Gut hormone profiles reflect a complex interplay of diet composition, enzymatic digestion, bile and intestinal secretions, gut motility and nutrient absorption rates, which can only be studied in whole animals and humans. Alterations in gastric emptying and gut motility have profound effects on gut hormone profiles following oral nutrient ingestion, but local intestinal perfusion bypassing the gastric pylorus and proximal alteration of motility can be achieved through direct cannulation in animals or a nasointestinal tube in humans.

## Fundamental signalling pathways in enteroendocrine cells

EECs share many fundamental properties with other endocrine cells, particularly pancreatic islet cell types with which they have common ancestry. Unlike islet cells, however, their polarised epithelial localisation means that their apical and basolateral surfaces are bathed in fluids of different composition and are differentially exposed to nutritional and other stimuli. Like pancreatic islet cells, EECs are electrically active, firing action potentials in response to stimuli such as glucose (Reimann *et al.* 2008). Their action potentials are mediated at least in part by voltage gated P/Q type calcium channels, as determined by the high expression of *Cacna1a* across human and mouse EECs, and the sensitivity of action potentials to ω-agatoxin-IVA (Goldspink *et al.* 2018*b*). In addition, however, EECs express T-type and L-type calcium channels (Goldspink *et al.* 2018*b*, 2020), with the latter likely underlying the calcium influx linked to hormone secretion, as blockers of L-type calcium channels such as nifedipine have been found to inhibit GLP-1 secretion in cell lines (Reimann *et al.* 2005), murine primary cultures (Rogers *et al.* 2011) and perfused mouse intestinal models (Kuhre *et al.* 2015).

Action potential firing in EECs is initiated when the membrane potential rises above a threshold level at which inward currents become amplified by low voltage activated channels, likely including T-type calcium channels. Reaching this threshold is dependent on the balance between depolarising currents (usually carried by sodium and calcium) and hyperpolarising currents (predominantly potassium). Although EECs express a variety of potassium channels, the resting potassium current is relatively small as determined from membrane resistance measurements (Reimann *et al.* 2008). This makes the cells readily responsive to small depolarising currents carried by, for example, TRP channels and electrogenic substrate transporters. The importance of the TRP channel repertoire of EECs remains incompletely characterised, even though a range of TRP channels have been identified in murine EECs (Shah *et al.* 2012, Emery *et al.* 2015, Goldspink *et al.* 2018*b*). TRP channels in EECs can respond directly to chemical stimuli such as mustard (TRPA1 (Emery *et al.* 2015)) or amplify second messenger signalling pathways downstream of Gαq-coupled receptors (e.g. TRPC3 downstream of FFAR1 in murine L-cells (Goldspink *et al.* 2018*b*); TRPM5 downstream of FFAR4 in STC-1 cells (Shah *et al.* 2012)), but some notable differences have been observed between *Trp* channel expression in EEC cell lines and native cells, as well as between humans and mice, suggesting different functionality.

Intracellular Ca^2+^ levels can be monitored in real time using genetically encoded Ca^2+^ indicators such as GCaMPs, or cell-permeable dyes such as fura2-AM (Dana *et al.* 2019). Elevation of intracellular Ca^2+^ levels has been observed across a range of EEC models in response to depolarising stimuli and GPCR activation, and provides a readout to validate the functional expression of Gαq-coupled receptors found at the RNA level (Goldspink *et al.* 2020). Bombesin, for example, increases Ca^2+^ in primary mouse L-cells via activation of the bombesin 2 receptor and is a correspondingly strong stimulus of GLP-1 secretion in perfused intestinal models (Svendsen *et al.* 2016).

cAMP is an intracellular second messenger signal produced by adenylate cyclase (AC) after the activation of Gαs-coupled receptors. In general, cAMP signals via protein kinase A (PKA) and exchange proteins activated by cAMP (Epac1/2) (Sassone-Corsi 2012), and can directly or indirectly modulate ion channels important for shaping endocrine cell electrical activity and calcium dynamics, including voltage-gated Ca^2+^ channels, hyperpolarisation-activated (HCN) ion channels and potassium channels. The accumulation of cAMP in EECs is generally accompanied by robust stimulation of hormone secretion. In L-cells, cAMP elevations promote release of hormone-containing vesicles by enhancing electrical activity, activating voltage-gated Ca^2+^ channels and potentiating Ca^2+^-dependent exocytosis (Simpson *et al.* 2007, Goldspink *et al.* 2018*b*), although the exact molecular targets of cAMP and consequent signalling events remain incompletely characterised. cAMP levels have been monitored in enteroendocrine cell lines and mouse and human L-cells using genetically encoded fluorescence resonance energy transfer (FRET)-based sensors (Klarenbeek *et al.* 2015), revealing for example that bile acids elevate cAMP through the Gαs-coupled bile acid receptor GPBAR1 (Brighton *et al.* 2015), and that monoacylglycerols elevate cAMP signalling downstream of GPR119 (Hodge *et al.* 2016). Reductions in cAMP below the baseline, downstream of Gαi-coupled receptor activation, are more difficult to monitor with FRET-based probes, but with prior cAMP elevation using IBMX or forskolin it has been possible to monitor cAMP decreases in EECs following treatment with Gαi-coupled receptor agonists such as somatostatin (Moss *et al.* 2012).

## Postprandial EEC sensing mechanisms

**Figure 1:**
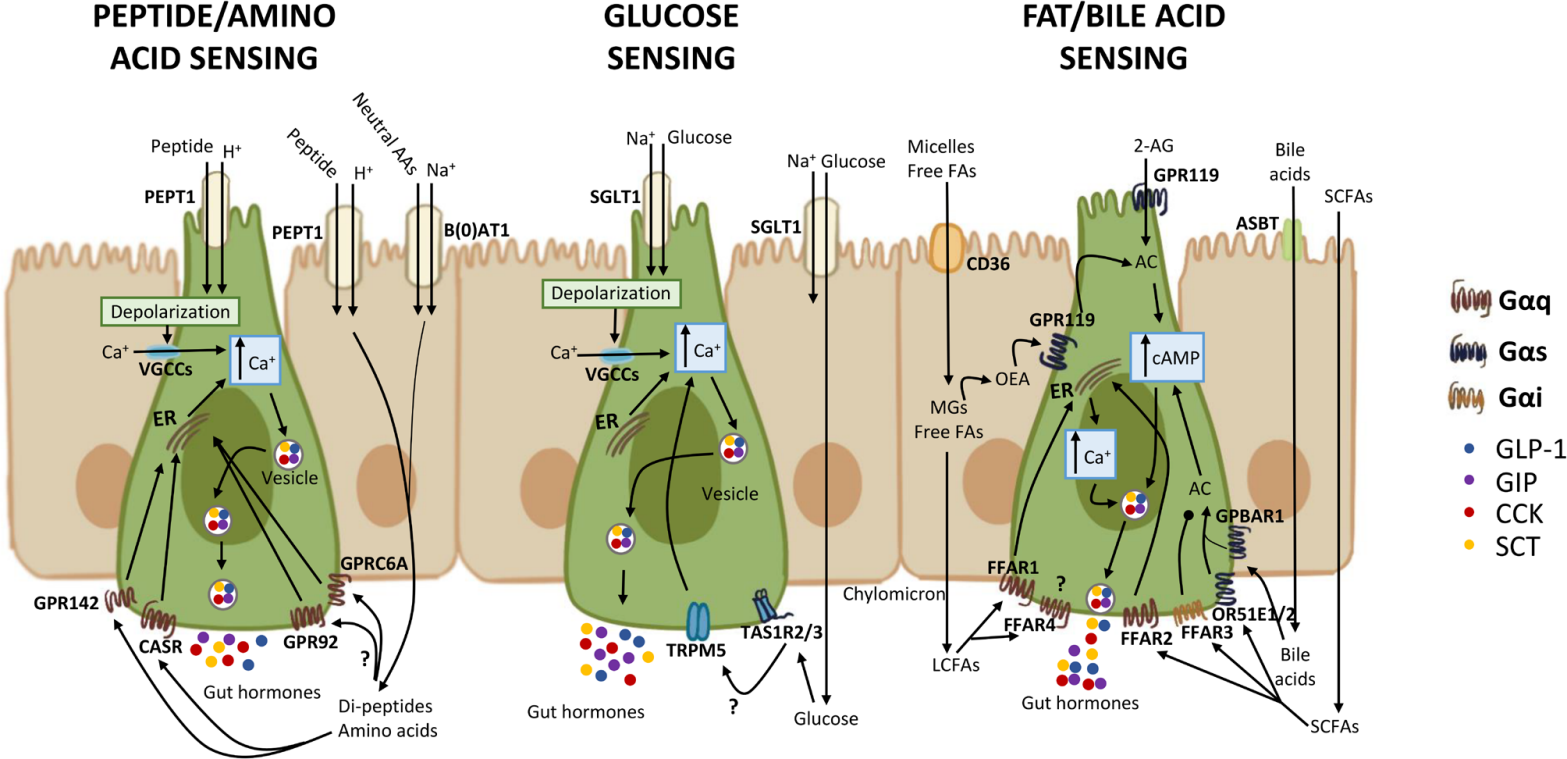
Nutrient-sensing mechanisms of intestinal hormone release from EECs. Peptides and amino acids, carbohydrates, fats, and bile salts can elicit responses either apically (top) or basolaterally (bottom). Peptides and amino acids are transported into EECs by PEPT1 (with H^+^) and B(0)AT (with Na^+^), respectively, whereas glucose is co-transported along with Na^+^ by SGLT1. This process causes membrane depolarisation and activates VGCCs, increasing intracellular calcium and therefore releasing gut hormones into the system. The same transporters are also located in the enterocytes so amino acids and glucose can be absorbed and reach the basolateral surface where different GPCRs are expressed, including CASR, GPR142, GPR93, GPRC6A and TAS1R2/3. Fatty acid and bile acid transporters are involved in their uptake across the brush border. Basolateral LCFAs can stimulate Gαq-coupled GPCRs FFAR1 and FFAR4, whereas SCFA can activate Gαi-coupled FFAR3, Gαi/q FFAR2 and Gαs OR51E1/2. Monoacylglycerols activate Gαs-coupled GPR119, provoking gut hormone secretion through increasing AC activity and cAMP levels. Abbreviations: peptide transporter 1 (PEPT1), neutral amino acid transporter 1 (B(0)AT), sodium-coupled glucose cotransporter 1 (SGLT1), cluster of differentiation (CD36), voltage-gated calcium channels (VGCCs), endoplasmic reticulum (ER), cyclic adenosine monophosphate (cAMP), adenylyl cyclase (AC), amino acids (AAs), long-chain fatty acids (LCFAs), short-chain fatty acids (SCFAs), oleoylethanolamide (OEA), 2-acylglycerol (2-AG), G-protein-coupled receptor (GPCR), calcium sensing receptor (CASR), transient receptor potential cation channel subfamily M member 5 (TRPM5), free fatty acid receptor 1-4 (FFAR1-4), G-protein-coupled bile acid receptor 1 (GPBAR1).

The major stimuli for postprandial gut hormone secretion are the three macronutrients – carbohydrates, fats and proteins. EEC stimulation occurs downstream of macronutrient digestion, and in most cases also of nutrient absorption (Fig. 1). Two broad classes of nutrient sensor have been identified in EECs: G-protein-coupled receptors (GPCR) and substrate transporters.

### GPCRs

The major classes of GPCR linked to stimulation of gut hormone release are either Gαq or Gαs coupled, resulting in elevation of Ca^2+^ and cAMP respectively, which synergise in stimulating GLP-1 secretion, implying convergence of cAMP and Ca^2+^ signals (Ekberg *et al.* 2016, Goldspink *et al.* 2018*b*). In the postprandial state, EECs are exposed to a cocktail of stimuli targeting a range of synergistic pathways, including glucose-dependent cell depolarisation (discussed below) and Gαs- and Gαq-coupled receptor activation, and it is likely that this confluence of signals arriving at individual EECs shapes the circulating gut hormone profiles. Gαi-coupled receptors typically lower cellular cAMP levels thereby reducing hormone secretion (Gribble & Reimann 2019), but unexpected coupling of the Gαi-coupled adrenoreceptor 2A to elevated Ca^2+^ via TRPC4 channel recruitment has been reported in enterochromaffin cells (Bellono *et al.* 2017) – another member of the EEC family – and it will be interesting to see whether TRP channels can also modify responses of incretin-secreting cells to other Gαi-coupled stimuli.

#### Fat and bile acid sensitive GPCRs

Triglyceride digestion, promoted by emulsification of fat with bile acids and digestion by intestinal lipases, releases fatty acids and 2-monoacylglycerols, which have stimulatory potential on EECs dependent on the properties of the individual fatty acid constituents. A minimum chain length of C12 has been widely reported for fatty acid stimulated GLP-1 and CCK elevation in humans, measured for example after intra-gastric or intraduodenal infusion (McLaughlin *et al.* 1999, Feltrin *et al.* 2004), which was also replicated in STC-1 cells (McLaughlin *et al.* 1998). Similar conclusions were drawn from studies involving oral ingestion of triglycerides in which fatty acids in the 1 and 3 positions, which are released by intestinal lipase digestion, were either long chain (oleic acid – olive oil) or medium chain (C8 – ‘dietary oil’). Only olive oil stimulated CCK release in this study, indicating the importance of long-chain fatty acids and not 2-mono-oleoylglycerol (2-OG) for CCK secretion. By contrast, GIP secretion increased with both oils but exhibited a bigger response to olive oil, suggesting additional stimulation by 2-OG; GLP-1 levels were elevated similarly by both oils (Mandoe *et al.* 2015).

Several GPCRs have been implicated as candidate EEC sensors for fatty acids, including FFAR1-4, GPR84, HCAR2 and olfactory receptors OR51E1 and E2, as discussed here.

FFAR1 (GPR40) responds to saturated and unsaturated long-chain fatty acids (Briscoe *et al.* 2003), and has been strongly implicated in gut hormone secretion across many model systems, validated by selective pharmacology and receptor knockout *in vitro* and *in vivo* (Gribble *et al.* 2016). Direct activation of murine CCK-producing I-cells by linoleic acid, for example was evident in calcium recordings, and was decreased in cells from *Ffar1* knockout mice (Liou *et al.* 2011*a*). The receptor is predominantly Gαq coupled, although second-generation agonists were also found to elevate cAMP levels (Hauge *et al.* 2017). A recent study has revealed that this is not due to Gαs coupling, as originally suggested, but to Gαq-dependent activation of adenylate cyclase 2 (Petersen *et al.* 2023). Several studies have reported that FFAR1 agonists induce insulin secretion, decrease body weight and food intake, and increase GLP-1 and GIP secretion *in vivo*. The second-generation FFAR1 agonist T3601386, for example, increased plasma GLP-1 and GIP levels after oral administration in wild-type but not *Ffar1* knockout mice, suggesting an FFAR1-dependent incretinotropic capacity (Ueno *et al.* 2019). Perfused intestine experiments have interestingly revealed that ligand access to FFAR1 on EECs is from the basolateral direction since it was observed that vascular, but not luminal administration of FFAR1 agonists stimulated GLP-1 release (Christensen *et al.* 2015). This is consistent with a number of physiological studies that had reported the importance of chylomicron formation for oral lipid-triggered gut hormone release (Lu *et al.* 2012).

FFAR4 (GPR120) is considered the second important long-chain fatty acid receptor, but evidence that it plays a role in gut hormone secretion is weaker than for FFAR1. It is a receptor for long chain, including polyunsaturated, fatty acids (Hirasawa *et al.* 2005), and is predominantly Gαq coupled, although Gαi and even Gαs coupling have been reported (Sundstrom *et al.* 2017). The potential for different FFAR4 agonists to exhibit bias towards either Gαi or Gαq likely contributes to the conflicting experimental results. In STC-1 cells, *Ffar4* knockdown reduced fatty acid triggered hormone secretion, supporting a signalling role in this cell line (Hirasawa *et al.* 2005). Fewer convincing results have been obtained in primary intestinal cultures and *in vivo*. In primary cultures, partial abrogation of lipid-triggered GLP-1 and GIP release was observed in *Ffar4* knockouts, particularly in combination with *Ffar1* KO (Ekberg *et al.* 2016, Reimann *et al.* 2020). *In vivo*, oil gavage in mice triggered CCK, GIP and GLP-1 release, of which the CCK and GIP but not GLP-1 responses were impaired in *Ffar4* knockout animals. Interestingly, the GIP response in *Ffar4* knockout mice was restored when impaired gallbladder contraction was reversed using CCK (Sankoda *et al.* 2017), suggesting that the loss of GIP release was indirect and due to a lack of CCK. Results from the same laboratory also reported that medium-chain fatty acids antagonise LCFA-triggered responses on FFAR4 (Murata *et al.* 2021) and suppress gallbladder contraction and digestive enzyme levels, suggesting a role for FFAR4 in CCK release. Supporting this idea, double knockout of *Ffar1* and *Ffar4* abolished the ability of mice to develop a learnt preference for high fat foods, via a pathway that was at least partially dependent on CCK signalling through the vagus nerve (Li *et al.* 2022). Our understanding of CCK release *in vivo* has lagged behind that of GIP and GLP-1 because of difficulties in measuring CCK *in vivo* due to frequent assay cross reactivities with gastrin, circulating at higher concentrations. However, newer mass-spectrometry based assays may circumvent this problem (Foreman *et al.* 2023). *Ffar4* is also highly expressed in goblet cells in the intestine and knockout mice have increased intestinal permeability which should also be taken into account when interpreting the physiology of this mouse model (Rubbino *et al.* 2022).

GPR84 is a medium-chain fatty acid receptor, recently suggested to play a role in GLP-1 secretion in mice. Lower GLP-1 plasma levels were observed in response to C10 fatty acids in *Gpr84* knockout animals, suggesting a GPR84-mediated GLP-1 secretory pathway which was backed up using antagonists in STC-1 cells (Nonaka *et al.* 2022). However, rather confusingly in the context of a potential activatory signal, GPR84 is Gαi-coupled, and in the light of the lack of responsiveness of human gut hormone release to medium-chain fatty acids *in vivo*, this receptor cannot be linked to a clear physiological role in the enteroendocrine system. The results rather highlight the importance of interpreting mouse and cell line data in the context of whether the implicated signalling machinery is also expressed and functional in human EECs.

OR51E1 and OR51E2 (mouse homologues, OLFR558 and OLFR78) are olfactory receptors responsive to carboxylic acids of chain lengths C4-C14 and C2-C3, respectively, and are coupled to Gαs signalling pathways (Jovancevic *et al.* 2017, Billesbolle *et al.* 2023). OLFR78 has been identified in mouse colonic PYY-secreting cells and linked to GLP-1 secretion from STC-1 cells and to plasma PYY levels in mice fed with fructo-oligosaccharides (Nishida *et al.* 2021). Corresponding with the murine data, OR51E2 was also differentially expressed in human organoid-derived GLP-1 secreting L-cells (Goldspink *et al.* 2020). OLFR558 has been identified in mouse enterochromaffin cells, where its activation by isovalerate has been linked to enhanced serotonin release (Bellono *et al.* 2017). The roles, if any, of either receptor in human incretin secretion remain uncertain.

FFAR2 (GPR43), FFAR3 (GPR41) and GPR109A (hydroxycarboxylic acid receptor HCAR2) are short-chain fatty acid (SCFA) receptors, potentially involved in detecting microbially generated SCFA in the distal intestine. Paradoxically for candidate activators of EEC secretion, all three receptors are Gαi/o coupled, although FFAR2 also exhibits Gαq coupling. Effects of SCFA on EEC number and function have been widely reported in murine models (Cani *et al.* 2007), primary cell cultures (Tolhurst *et al.* 2012) and intestinal organoids (Petersen *et al.* 2014), although SCFA did not trigger a convincing acute GLP-1 secretory response in perfused rodent intestine (Christiansen *et al.* 2018) and our laboratory was unable to demonstrate activity of the pathway in GLUTag cells, which lack *Ffar2* expression. GLP-1 secretion triggered by acetate and propionate was impaired in mouse primary colonic cultures derived from *Ffar2* or *Ffar3* knockout mice (Tolhurst *et al.* 2012), and effects of butyrate on expression of *Pyy* in human and mouse has been partially linked to FFAR2 (Larraufie *et al.* 2018). Although FFAR3 is Gαi coupled, a selective FFAR3 agonist AR420626 was found to significantly enhance GLP-1 release (Nøhr *et al.* 2013), raising the possibility that additional important signalling pathways may be recruited downstream of these (and potentially other Gαi-coupled) receptors. One or more of these SCFA receptors may also be responsible for the observed pertussis toxin-sensitive inhibition of GLP-1 release by ketone bodies such as betahydroxybutyrate (Wallenius *et al.* 2020). The importance of SCFA as GLP-1 secretagogues has recently been challenged in a study demonstrating that GLP-1 responses shortly after lactulose ingestion had little to do with its fermentation and rather reflect the arrival of a hyperosmotic load in the distal small intestine (Christiansen *et al.* 2022); however, a study inhibiting proximal sucrose digestion with acarbose reported a correlation between H_2_-exhalation (a measure of intestinal sucrose fermentation) and increased GLP-1 secretion 2–3 h after sucrose ingestion (Seifarth *et al.* 1998), supporting a role for SCFA-sensing in humans.

GPR119 is a Gαs-coupled receptor responsive to monoacylglycerols, and is highly expressed in L- and K-cells (Hassing *et al.* 2016, Mandoe *et al.* 2018). As monoacylglycerols are produced alongside fatty acids by intestinal triglyceride digestion, GPR119 has potential to be activated in tandem with FFAR1 in EECs. Indeed, GPR119 has been reported to synergise with FFAR1 to release GLP-1 in primary cultures (Ekberg *et al.* 2016). *In vivo* studies have highlighted the importance of this receptor for fat-dependent GLP-1 secretion after meal ingestion. Thus, mice lacking GPR119 in L-cells exhibited severe blunting of GLP-1 responses after a gavage oil challenge (Hodge *et al.* 2016), and in humans fed either olive oil or dietary oil (which is digested to two C8 fatty acids and a monooleoylglycerol) the GLP-1 response was similar to both oils highlighting the importance of the monoacylglycerol moiety (Mandoe *et al.* 2015). In this human study, GIP but not CCK release was also concluded to respond to the GPR119 component.

GPBAR1 (G-protein-coupled bile acid receptor 1), also known as TGR5, is activated by bile acids, which are released into the intestinal lumen by CCK-regulated gallbladder contraction. As they form micelles with fatty acids and monoglycerides to promote absorption, they deserve consideration alongside the lipid stimuli. GPBAR1 is a Gαs-coupled receptor that is highly expressed in L-cells and strongly implicated in triggering secretion of GLP-1 in different model systems including cell cultures and perfused intestinal models (Goldspink *et al.* 2018*b*, Kuhre *et al.* 2018). Similar to GPR119, agonism of GPBAR1 acts synergistically with Gαq receptors like FFAR1 to stimulate human and mouse L-cells *in vitro* (Goldspink *et al.* 2018*b*). As GPBAR1 is also expressed in brown adipose tissue, where its activation enhances energy expenditure (Thomas *et al.* 2009), this receptor was thought to be an interesting candidate drug target for treating metabolic disease until it was shown that its activation promotes gallbladder filling through relaxation of the gallbladder smooth muscle (Li *et al.* 2011). This has raised the question of whether non-absorbable GPBAR1 agonists could be developed that would target EECs, and by not entering the circulation would circumvent off-target effects. A number of studies, however, have concluded that bile acid absorption across the epithelium is essential for luminal ligands to reach GPBAR1 receptors, which appear to be functionally accessible only from the basolateral direction, making it unlikely that a simple non-absorbable strategy would promote selective targeting of EECs (Brighton *et al.* 2015, Kuhre *et al.* 2018); limited systemic bioavailability by firstpass clearance in the liver, might, however, be a promising strategy.

#### Sweet and bitter taste receptors

TAS1R GPCRs have been implicated in detection of sweet tasting substances by the tongue, but there is little compelling evidence that they act as direct sensors of sugar ingestion in EECs. The oral sweet taste receptor is a heterodimer of the taste 1 receptor family members TAS1R2 and TAS1R3, coupled to downstream signalling through alpha-gustducin, phospholipase C beta 2 and TRPM5. Although some reports have identified TAS1R subunits in EECs by immunostaining (Jang *et al.* 2007), this is controversial and is not reproduced by transcriptomic analysis, which has failed to identify EEC expression of *Tas1r2* or other important sweet-taste signalling components in a number of studies (Reimann *et al.* 2008, Goldspink *et al.* 2020). Although a *Trpm5*-expressing cell population was identified in the gut, its transcriptomic signature was not found to be typical of EECs (Bezencon *et al.* 2008). Despite our inability to stimulate secretion of GLP-1 with TAS1R2/3 agonists (Reimann *et al.* 2008), other groups have reported effects of artificial sweeteners on EEC secretion (Buchanan *et al.* 2022). Some *in vivo* studies reported clinical evidence that blocking sweet taste receptor using lactisole attenuated glucose-induced release of GLP-1 and PYY but not CCK, suggesting that sweet taste receptor signalling could be involved in glucose-mediated GLP-1 secretion, although the receptor alone is not responsible for GLP-1 secretion (Gerspach *et al.* 2011). However, other human studies have failed to demonstrate release of GIP or GLP-1 using artificial sweeteners as the stimulus (Ma *et al.* 2009, Kuhre *et al.* 2015), supporting our view that TAS1R2/3 does not play an important role in sugar sensing in incretin secreting cells. A recent study revealed that lactisole did not affect the erythritol and d-allulose-induced secretion of GLP-1, CCK and PYY in humans, concluding that the secretion induced by those artificial sweeteners is not mediated by TAS1R2/TAS1R3 in the gut (Teysseire *et al.* 2022).

There is an increasing interest in the role of TAS2R ‘bitter taste’ receptors in relation to gut hormone secretion. In humans there are 25 known members, some of which respond to a broad spectrum of ligands, whereas others have more specific ligand requirements (Descamps-Sola *et al.* 2023). Some TAS2Rs are broadly expressed in the gut epithelium, whilst others are enriched in EECs, and one recent report observed a small stimulation of GLP-1 and CCK release (yet no GIP response) within 30 min of intraduodenal quinine application (Rezaie *et al.* 2023). As the authors discuss, care should, however, be taken to conclude that this was TAS2R mediated, given that quinine can also affect other proteins, such as ion channels. By contrast, another recent study failed to observe an effect on CCK secretion when quinine or denatonium benzoate were delivered intraduodenally (Verbeure *et al.* 2021) and a recent systematic literature review concluded that TASR2s likely play a role in ghrelin and motilin secretion, but failed to find conclusive evidence for significant effects of bitter stimulants on GLP-1 or PYY release (Hassan *et al.* 2023).

### Protein sensing GPCRs in EECs

Protein sensing in EECs occurs downstream of protease digestion into oligopeptides and amino acids. A number of GPCRs have been proposed to play a role in EEC peptide and amino acid sensing, including the calcium sensing receptor (CASR), umami taste receptors (TAS1R1/TAS1R2), GPR142, GPR92 and GPRC6A. Agonism of GPR93, GPR142 and the umami taste receptor with specific agonists or allosteric modulators did not, however, increase GLP-1 secretion in the perfused rat intestinal model (Modvig *et al.* 2021). A comprehensive analysis of amino acid-triggered secretion in perfused rat intestine identified that arginine, phenylalanine and tryptophan stimulated GLP-1 release when infused vascularly, whereas only valine and phenylalanine stimulated secretion when infused intraluminally (Modvig *et al.* 2021). The mechanism of valine sensing in this model has not yet been characterised.

CASR is classically activated by aromatic amino acids such as phenylalanine and tryptophan, and possibly also by short peptides. It is a complicated Gαq-coupled receptor with a variety of ligand binding sites on its N-terminal venus fly-trap domain, including for calcium ions (Leach *et al.* 2020). It is highly expressed by many EECs and model cell lines, including cells secreting CCK, GIP and GLP-1. Calcium responses in primary I-cells and CCK secretion triggered by phenylalanine were abolished in cells from *Casr* knockout mice (Liou *et al.* 2011*b*), and in the perfused rat small intestinal model, CASR agonists seemed to access basolaterally located receptors to trigger GLP-1 release since Calindol, a positive allosteric modulator of CASR, stimulated GLP-1 secretion when infused vascularly, but not luminally (Modvig *et al.* 2019). Whereas both phenylalanine and tryptophan enhanced GLP-1 secretion when perfused from the vascular side, only phenylalanine was effective from the luminal direction, likely explained by the robust transepithelial absorption of phenylalanine but not tryptophan (Modvig *et al.* 2021).

GPR142 is a Gαq-coupled GPCR that senses aromatic amino acids such as tryptophan and phenylalanine. In mouse models, the GPR142 agonist C-22 increased plasma GIP, GLP-1 and CCK levels after oral administration, and in each case this response was lost in *Gpr142* knockout mice (Rudenko *et al.* 2019). However, plasma GLP-1 and GIP responses to a protein diet challenge were not impaired in *Gpr142* knockout mice (Rudenko *et al.* 2019), and an alternative agonist LY3201143 did not enhance GLP-1 levels in the perfused rat intestine (Modvig *et al.* 2021), raising uncertainties about the physiological importance of GPR142 for EEC protein sensing.

GPRC6A is a Gαq-coupled receptor responsive to basic amino acids such as arginine and ornithine (Wellendorph *et al.* 2005). It is expressed in GLUTag cells where it was found to underlie responses to ornithine, but its physiological importance was questioned because it could not be detected in native mouse L-cells (Oya *et al.* 2013). As plasma GLP-1 responses triggered by oral administration of L-arginine or L-ornithine were not impaired in *Gprc6a* knockout mice (Clemmensen *et al.* 2017), a role, if any, for GPRC6A in the enteroendocrine system remains uncertain.

GPR92(also known as GPR93 or LPAR5) is highly expressed in the mouse gastrointestinal tract (Symonds *et al.* 2015). Some studies have proposed the involvement of this receptor in CCK and GLP-1 secretion in response to peptones and food peptides in STC-1 cells (Choi *et al.* 2007, Santos-Hernandez *et al.* 2023). However, *Gpr93* expression was not detected in colonic L-cells, and peptone-triggered GLP-1 secretion was not altered in primary intestinal cultures from *Gpr93*-deficient mice, suggesting that it may not contribute significantly to amino acid sensing in L-cells (Diakogiannaki *et al.* 2013).

## Transporters and ion channels

SGLT1 (sodium-glucose transporter 1, *Slc5a1*) is co-expressed in primary L-cells with other candidate glucose-sensing machinery including ATP-sensitive K^+^ (K_ATP_) channel subunits (KIR6.2, SUR1), glucose transporter 2 (GLUT2 encoded by *Slc2a2*) and glucokinase (GCK). Whereas pancreatic beta cells utilise a metabolism dependent K_ATP_ channel pathway for glucose sensing, a multitude of studies have highlighted SGLT1 as the critical glucose sensor in K- and L-cells. Absorption of monosaccharide substrates at the luminal membrane by SGLT1 carries positively charged Na^+^ ions, inducing membrane depolarisation and hormone release (Parker *et al.* 2012). The role of SGLT1 as the luminal glucose sensor has been validated in cell lines, primary cells and perfused intestinal models (Gorboulev *et al.* 2012, Kuhre *et al.* 2015). Plasma GIP responses to oral glucose, and early plasma GLP-1 elevations are abolished in *Sglt1* knockout mice (Gorboulev *et al.* 2012). Delayed glucose-triggered GLP-1 but not GIP responses have been observed after SGLT1 inhibition and in *Sglt1* knockout mice (Powell *et al.* 2013), but the underlying pathway remains unclear. Apart from its importance for incretin secretion, it is now clear that SGLT1 is crucial for the development of post-ingestive sugar preference (Li *et al.* 2022), which likely involves enteroendocrine components (Liu & Bohorquez 2022).

Peptide transporter 1 (PEPT1, *Slc15a1*) has been implicated as a critical step for GLP-1 secretion triggered by luminal peptones. It is an electrogenic transporter that couples uptake of di- and tripeptides to the influx of H^+^ ions, and is capable of directly depolarising EECs and triggering GLP-1 release as demonstrated in primary mouse L-cells stimulated with the non-metabolisable substrate Gly-Sar (Diakogiannaki *et al.* 2013). However, the PEPT1 inhibitor 4-aminomethylbenzoic acid (4-AMBA) did not impair peptone-triggered GLP-1 release in primary cultures, and in STC-1 cells PEPT1-dependent H^+^ currents were only large enough to elicit membrane depolarisation following heterologous *Pept1* overexpression (Matsumura *et al.* 2005). In perfused rat small intestine, GLP-1 responses to luminally infused peptones were impaired by 4-AMBA or by inhibition of CASR, and it was concluded that the predominant role of PEPT1 was to deliver peptide substrates across the epithelium where they would be detected by basolaterally located CASR (Modvig *et al.* 2019). Whether H^+^ currents carried PEPT1 contribute significantly to EEC stimulation *in vivo* remains unestablished.

B(0)AT-1 (Slc6a19) is another electrogenic amino-acid transporter located on the intestinal brush border, with unclear roles in EEC secretion. *Slc6a19* knockout mice showed altered GIP and GLP-1 responses compared to their wild type littermates, which at least for GLP-1 could reflect increased delivery of unabsorbed nutrients to the more distal intestine (Jiang *et al.* 2015). B(0)AT1 inhibitors are under investigation as a potential strategy to treat type 2 diabetes (Cheng *et al.* 2017, Joharapurkar *et al.* 2022), although the mechanism has not been elucidated.

## Other stimuli shaping postprandial incretin secretion

In addition to direct nutrient sensing by K- and L-cells, paracrine crosstalk between EEC further shapes postprandial hormone secretion. Somatostatin (SST) is an intestinal hormone, secreted from D-cells, that suppresses the release of intestinal and pancreatic hormones, including GIP and GLP-1 (Jepsen *et al.* 2019). Several studies have demonstrated the inhibitory effect of SST on GIP secretion in enteroendocrine enriched cultures and in mixed epithelial cultures (Kieffer *et al.* 1994, Moss *et al.* 2012). As SST release, in turn, is stimulated by GIP and GLP-1 (Holst *et al.* 1983), this suggests a complex crosstalk between these cell types in the intact epithelium. Incretin hormones also stimulate secretion of serotonin (5-HT) from epithelial enterochromaffin cells (Lund *et al.* 2020, Tough *et al.* 2023), and 5-HT-4 receptor activation has been shown to increase L-cell number in murine and human organoids and in mice (Lund *et al.* 2020), adding a further level of complexity. CCK has been shown to enhance GIP secretion in mice through an indirect pathway involving stimulation of the exocrine pancreas and gallbladder, thereby enhancing proximal postprandial lipid digestion and generating local lipid stimuli to K-cells in this region (Murata *et al.* 2021). Other hormones/factors present in the intestine, either produced locally or released by enteric and/or autonomic nerves, have also been shown to modulate incretin secretion – examples include inhibition of GIP but not GLP-1 secretion by endocannabinoids through CB1 receptors in primary murine intestinal cultures (Moss *et al.* 2012), and inhibition of the release of both incretins by galanin in these cultures (Psichas *et al.* 2016). Gastrin-releasing peptide stimulates GLP-1 but not GIP release in mice (Roberge *et al.* 1996, Svendsen *et al.* 2016) and vasopressin (Pais *et al.* 2016*b*) and angiotensin (Pais *et al.* 2016*a*) have been shown to stimulate GLP-1 release in primary murine and human epithelial cultures.

## Future perspectives and conclusion

The incretin hormones GLP-1 and GIP have critical roles in the stimulation of glucose-induced insulin secretion, appetite regulation, food intake and body weight after their nutrient-induced secretion from the gut (Seino & Yamazaki 2022). A number of GLP-1 receptor agonists are clinically used and highly effective for treating type 2 diabetes and obesity (Davies *et al.* 2021) and dual incretin receptor agonists targeting both GIP and GLP-1 receptors appear to offer even better metabolic and body weight benefits (Campbell *et al.* 2023). Reports consistently demonstrate that postprandial GLP-1 and PYY secretion is enhanced after bariatric surgery, and evidence that these hormones contribute to postsurgical weight loss and improved glucose tolerance (Dirksen *et al.* 2013, Svane *et al.* 2016), have highlighted the translational potential of targeting the enteroendocrine system pharmacologically. Interestingly postprandial GIP responses after Roux-en-Y gastric bypass (RYGB) have inconsistently been reported as elevated, decreased or unchanged (Douros *et al.* 2019, Moffett *et al.* 2021), which might be a consequence of altered nutrient delivery to the proximal small intestine affecting the timing of GIP responses after RYGB and sleeve gastrectomy (Svane *et al.* 2019). The receptors and pathways involved in physiological nutrient sensing, described in this review, are, however, obvious candidate drug targets to modulate release of both incretins, but which of these will have robust effects on gut hormone secretion *in vivo* remain to be determined. It is possible that high levels of gut hormone release will only be achieved when synergistic pathways in EECs are recruited either by combining drugs targeting more than one receptor or by taking advantage of dietary stimuli to recruit synergistic signalling pathways in EECs. It is also not yet proven that therapeutic activation of GPCRs in EECs will trigger sufficient gut hormone release to produce significant metabolic benefits or satiation in humans. It has become clear from the transcriptomic overlap between EEC populations that it would likely be difficult to stimulate release of a single gut hormone such as GLP-1 or PYY without additionally increasing secretion of GIP, CCK and neurotensin, but the success of poly-receptor pharmacy against gut hormone receptors would suggest that increasing release of several hormones in parallel could itself offer therapeutic advantages.

Future research in this field promises to provide us with a better understanding of the mechanisms that regulate gut hormone secretion and to identify alternative strategies for treating type 2 diabetes and obesity.

## Declaration of interest

The authors declare that there is no conflict of interest that could be perceived as prejudicing the impartiality of this thematic review.

## Funding

Research in the Gribble/Reimann laboratory is funded by grants from Wellcome (220271/Z/20/Z) and MRC (MRC_MC_UU_12012/3). MSH holds a Martin Escudero fellowship.
